# A *Ganoderma lucidum* Polysaccharide-Modified Nanoadjuvant Elicits Potent Cellular Immunity for Hepatitis B Vaccine

**DOI:** 10.3390/vaccines14090779

**Published:** 2026-09-06

**Authors:** Zhe Zhai, Jiansong You, Xuhan Liu

**Affiliations:** 1 Guangdong Provincial Key Laboratory of Chinese Medicine Ingredients and Gut Microbiomics, Institute for Inheritance-Based Innovation of Chinese Medicine, Marshall Laboratory of Biomedical Engineering, School of Pharmacy, School of Basic Medical Sciences, Shenzhen University Medical School, Shenzhen University, Shenzhen 518055, China; 2 R&D Center, Aim Honesty Biopharmaceutical Co., Ltd., Dalian 116620, China

**Keywords:** hepatitis B vaccine, HBsAg, nanoadjuvant, *Ganoderma lucidum* polysaccharide, cellular immunity, Th1/Th2 balance

## Abstract

Background: Aluminum-adjuvanted hepatitis B vaccines effectively prevent primary HBV infection but predominantly favor humoral immunity and have limited capacity to induce strong cellular responses. To improve immune-response quality, we developed a *Ganoderma lucidum* polysaccharide-modified hybrid nanoparticle adjuvant (GHNP) for hepatitis B surface antigen (HBsAg) vaccination. Methods: Cationic PEG-PCL/DOTAP hybrid nanoparticles (HNPs) were surface-modified with *Ganoderma lucidum* polysaccharide (GLP) at different mass ratios and characterized by particle size, polydispersity index, zeta potential, and transmission electron microscopy. Antigen uptake, RAW264.7 cell viability, MHC II expression, and TLR2/4-associated TNF-α secretion were evaluated in vitro. C57BL/6 mice were immunized intramuscularly with PBS, HBsAg, HBsAg + Aluminum, HBsAg@HNP, or HBsAg@GHNP6 on Days 0, 14, and 28. HBsAg-specific antibodies, splenocyte activation, proliferation, and cytokine secretion were assessed. Results: GHNP6 showed suitable physicochemical properties, acceptable cytocompatibility at concentrations up to 10 μg·mL^−1^, and efficient model-antigen uptake. GHNP increased MHC II expression in RAW264.7 cells, and TLR2/4 inhibition reduced nanoparticle-associated TNF-α secretion, supporting involvement of these pathways in immune activation. In vivo, HBsAg@GHNP6 induced robust HBsAg-specific IgG responses and a higher IgG2a/IgG1 ratio than HBsAg@HNP and HBsAg + Aluminum. It also produced the highest splenocyte proliferation after HBsAg restimulation, with lower IL-6 secretion than HBsAg + Aluminum and moderately increased IFN-γ compared with PBS and free HBsAg. Conclusions: GHNP integrates nanoparticle-assisted antigen delivery with GLP-mediated immunomodulation and represents a promising strategy for enhancing cellular immune responses while maintaining humoral immunity in HBsAg vaccination.

## 1. Introduction

Hepatitis B virus (HBV) infection remains a major global public health challenge. According to the World Health Organization (WHO), approximately 296 million people worldwide are chronically infected with HBV [1], and over 800,000 deaths occur annually due to HBV-related liver diseases [2]. One of the most effective strategies for preventing HBV infection is vaccination with recombinant subunit vaccines based on hepatitis B surface antigen (HBsAg). Since its introduction in 1982, this vaccine has been widely administered globally and has significantly reduced the incidence of new HBV infections [3]. However, most conventional recombinant HBsAg vaccines rely on aluminum salts, particularly aluminum hydroxide, as classical adjuvants to enhance humoral immune responses. Although alum adjuvants have been clinically used for nearly a century due to their proven safety profile, low cost, and robust antibody-enhancing effects, their immune activation pattern suffers from an inherent structural limitation: alum is a classic TLR-independent, purely Th2-biased adjuvant that efficiently induces humoral immunity (high titers of neutralizing antibodies, predominantly IgG1) but fails to activate Th1-type cellular immune responses [4]. It does not induce CD8^+^ cytotoxic T lymphocyte (CTL) responses nor effectively stimulate the secretion of type I cytokines such as IFN-γ [5]. This immunological bias means that while alum-adjuvanted hepatitis B vaccines can prevent primary infection, they lack the ability to eliminate established intracellular latent infections [6]. Moreover, protection rates are notably lower in “hyporesponsive/non-responsive” populations, including the elderly, obese individuals, and immunocompromised patients. Therefore, breaking the “Th2-only” immune ceiling of alum adjuvants and developing novel adjuvant systems capable of simultaneously eliciting both humoral and Th1-type cellular immune responses represent a critical unmet need in the field of HBV vaccine development.

To move beyond sole reliance on alum adjuvants, researchers over the past two decades have extensively explored two main strategies: (1) molecular adjuvants or pattern recognition receptor (PRR) agonists [5] (e.g., MPL/TLR4 agonist, CpG/TLR9 agonist, cGAMP/STING agonist) combined with aluminum salts (such as GSK’s AS04 system) [7], which forcibly drive Th1 skewing through exogenous immune-stimulatory signals; and (2) nanocarrier platforms serving as antigen delivery systems/physical adjuvants, leveraging their controllable particle size, surface charge, antigen encapsulation/adsorption, and sustained-release properties to improve antigen-presenting cell (APC) uptake efficiency and lymphoid organ targeting [8].

However, nanocarriers generally exhibit limited capacity for modulating immune subtypes. The physicochemical adjuvant effects of nanocarriers primarily arise from non-specific mechanisms such as cell membrane interactions and enhanced APC uptake [9]. While these carriers may contribute to overall antibody enhancement, they often fail to precisely regulate the Th1/Th2 balance in the manner of natural immune recognition molecules [10]. In other words, nanocarriers can serve as better delivery vehicles [11], but they rarely function autonomously as immune steering wheels. This explains why many nano-adjuvants achieve only modest gains in total IgG levels yet remain passively dependent on the intrinsic properties of the antigen regarding the type of immune response elicited, failing to systematically overcome the gap in cellular immunity left by alum adjuvants.

Viral surfaces typically display glycoproteins that are specifically recognized by cells and trigger potent antiviral responses [12]. *Ganoderma lucidum* is a well-known medicinal mushroom that has long been used as a traditional medicinal [13] and dietary resource [14]. Its polysaccharides have attracted considerable attention because of their favorable biocompatibility and broad immunomodulatory activities [15]. In this context, natural immunomodulatory polysaccharides—particularly *Ganoderma lucidum* polysaccharide (GLP)—present a highly promising adjuvant candidate. As a core active component of traditional medicinal fungi in China, GLP has been extensively demonstrated to possess multi-target immune-activating capabilities, exhibiting Th1/Th2 bidirectional immunomodulatory effects and even Th1-biasing potential in animal models [16,17]. More importantly, GLP is naturally derived, exhibits low toxicity, and offers significant safety advantages over synthetic adjuvants, aligning with the trend toward “green adjuvant” development [18]. These characteristics motivated our selection of GLP as a natural immunomodulatory component to complement the antigen-delivery function of the nanoparticle carrier. However, to date, few studies have applied GLP as an adjuvant for hepatitis B vaccines or systematically evaluated its regulatory effects on immune response types compared with alum adjuvants.

**Figure 1:**
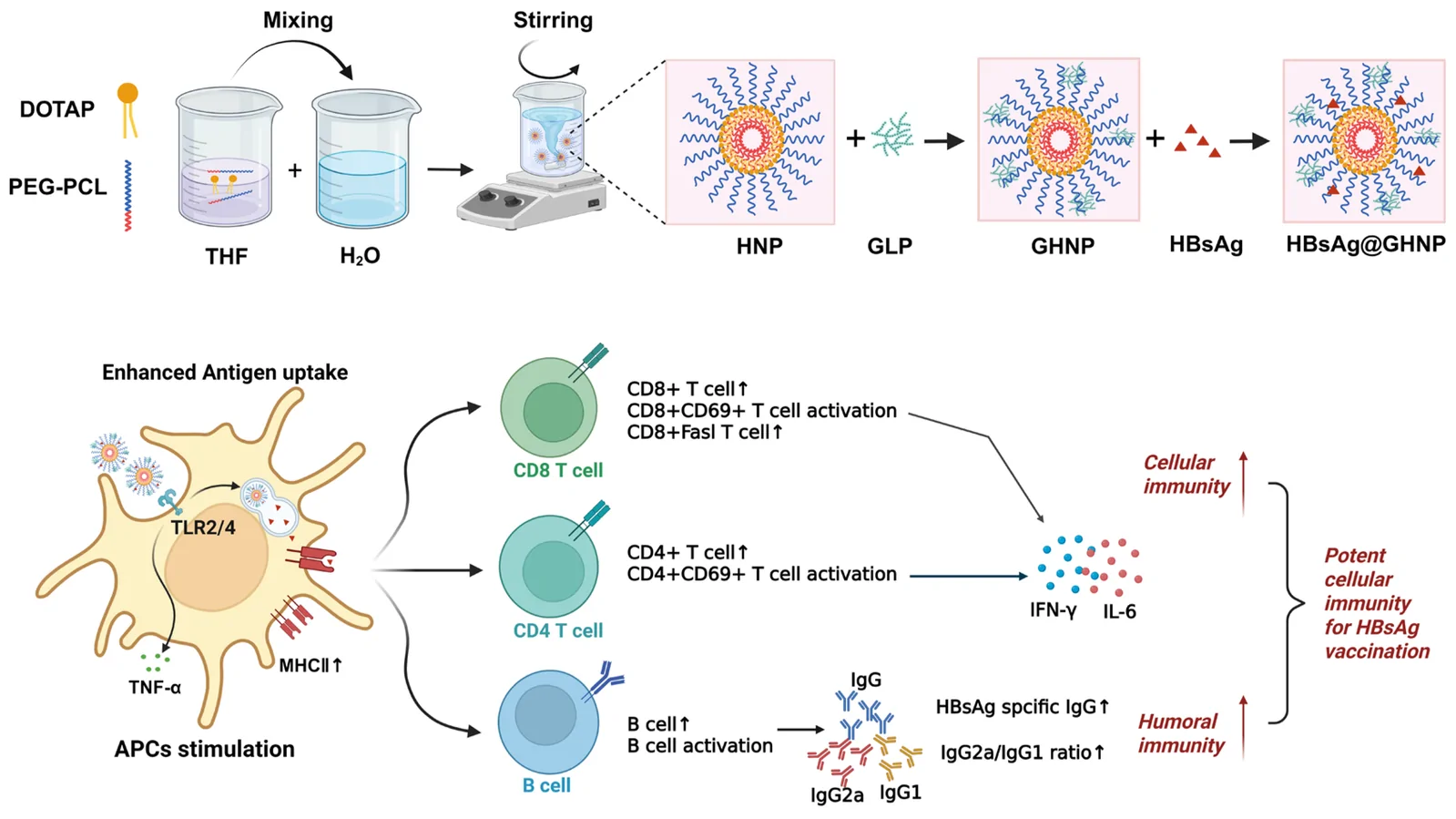
Schematic illustration of HBsAg@GHNP preparation and its immune-potentiating mechanism for enhanced humoral and cellular responses.

Based on the above gaps, this study proposes the following scientific hypothesis: by constructing a nanoparticle (HNP) composed of poly(ethylene glycol) -poly(ε-caprolactone) (PEG-PCL)/1,2-dioleoyl-3-trimethylammonium-propane (DOTAP) and loading GLP onto its surface via adsorption/modification to form a GLP-HNP composite adjuvant system, the carrier can efficiently deliver the hepatitis B antigen while GLP imparts its inherent immunomodulatory “program” to the nanovaccine interface. This would endow the system with active Th1/Th2 balance regulation capability, thereby breaking the immune subtype silence of bare HNP carriers and the Th2 monopoly of alum adjuvants, to achieve a distinct shift in immune response type compared to alum. Specifically, using Hepatitis B surface antigen (HBsAg) as the model antigen, this study investigates the GLP-modified HNP nano-adjuvant by focusing on: (1) changes in total antigen-specific IgG and IgG1/IgG2a subclass ratios, serving as indirect but classic markers of Th1/Th2 polarization; and (2) splenocyte proliferation and cytokine profiles. This study aims to develop a novel adjuvant capable of regulating the type (quality) rather than merely the magnitude (quantity) of immune responses (Figure 1).

## 2. Materials and Methods

### 2.1. Materials

*Ganoderma lucidum* (identified by the Institute of Microbiology, Chinese Academy of Sciences) was obtained from Dayao County, Chuxiong Yi Autonomous Prefecture, Yunnan Province, China. Poly(ethylene glycol)_5k_-poly(ε-caprolactone)_13k_ (PEG_5k_-PCL_13k_) and fluorescein isothiocyanate-conjugated bovine serum albumin (FITC-BSA) were purchased from Guangzhou Weihua Biotechnology Co., Ltd. (Guangzhou, China). Hepatitis B surface antigen (HBsAg) was provided by Aim Honesty Biopharmaceutical Co., Ltd. (Dalian, China). Aluminum adjuvant was purchased from Miragen Biotechnology Co., Ltd. (Shanghai, China). 1,2-dioleoyl-3-trimethylammonium-propane (DOTAP) and ethyl (6R)-6-[N-(2-chloro-4-fluorophenyl)sulfamoyl]cyclohex-1-ene-1-carboxylate (Resatorvid, TAK-242) were obtained from Shanghai Macklin Biochemical Technology Co., Ltd. (Shanghai, China). Tetrahydrofuran (THF) and 5H-benzocycloheptene-8-carboxylic acid, 3,4,6-trihydroxy-2-methoxy-5-oxo-, hexyl ester (CU-CPT22) were purchased from Shanghai Aladdin Biochemical Technology Co., Ltd. (Shanghai, China). Cell culture reagents, including 1× phosphate-buffered saline (PBS), penicillin-streptomycin solution, Dulbecco’s modified Eagle medium (DMEM), and trypsin, were acquired from Gibco (Waltham, MA, USA). Fetal bovine serum (FBS) was procured from NEWZERUM (Auckland, New Zealand). The Cell Counting Kit-8 (CCK-8) was supplied by Dalian Meilun Biotechnology Co., Ltd. (Dalian, China). Fluorescein isothiocyanate-labeled major histocompatibility complex class II antibody (FITC-MHC II), and mouse ELISA kits for tumor necrosis factor-alpha (TNF-α), interleukin-6 (IL-6), and interferon-gamma (IFN-γ) were purchased from Elabscience Biotechnology Co., Ltd. (Wuhan, China). APC Rat Anti-Mouse CD8a, FITC Rat Anti-Mouse CD4, PerCP-Cy™5.5 Rat Anti-Mouse CD19, and PE Rat Anti-Mouse CD69 antibodies were purchased from BD Biosciences (San Jose, CA, USA). PE anti-mouse CD178 (FasL) antibody was purchased from BioLegend (San Jose, CA, USA). Reagents for detecting HBsAg-specific IgG, IgG1, and IgG2a were purchased from Jiangsu Meimian Industrial Co., Ltd. (Yancheng, China). Mouse Spleen Lymphocyte Isolation Solution Kit was supplied by Beijing Solarbio Science & Technology Co., Ltd. (Beijing, China).

*Ganoderma lucidum* (identified by the Institute of Microbiology, Chinese Academy of Sciences) was obtained from Dayao County, Chuxiong Yi Autonomous Prefecture, Yunnan Province, China. Poly(ethylene glycol)_5k_-poly(ε-caprolactone)_13k_ (PEG_5k_-PCL_13k_) and fluorescein isothiocyanate-conjugated bovine serum albumin (FITC-BSA) were purchased from Guangzhou Weihua Biotechnology Co., Ltd. (Guangzhou, China). Hepatitis B surface antigen (HBsAg) was provided by Aim Honesty Biopharmaceutical Co., Ltd. (Dalian, China). Aluminum adjuvant was purchased from Miragen Biotechnology Co., Ltd. (Shanghai, China). 1,2-dioleoyl-3-trimethylammonium-propane (DOTAP) and ethyl (6R)-6-[N-(2-chloro-4-fluorophenyl)sulfamoyl]cyclohex-1-ene-1-carboxylate (Resatorvid, TAK-242) were obtained from Shanghai Macklin Biochemical Technology Co., Ltd. (Shanghai, China). Tetrahydrofuran (THF) and 5H-benzocycloheptene-8-carboxylic acid, 3,4,6-trihydroxy-2-methoxy-5-oxo-, hexyl ester (CU-CPT22) were purchased from Shanghai Aladdin Biochemical Technology Co., Ltd. (Shanghai, China). Cell culture reagents, including 1× phosphate-buffered saline (PBS), penicillin-streptomycin solution, Dulbecco’s modified Eagle medium (DMEM), and trypsin, were acquired from Gibco (Waltham, MA, USA). Fetal bovine serum (FBS) was procured from NEWZERUM (Auckland, New Zealand). The Cell Counting Kit-8 (CCK-8) was supplied by Dalian Meilun Biotechnology Co., Ltd. (Dalian, China). Fluorescein isothiocyanate-labeled major histocompatibility complex class II antibody (FITC-MHC II), and mouse ELISA kits for tumor necrosis factor-alpha (TNF-α), interleukin-6 (IL-6), and interferon-gamma (IFN-γ) were purchased from Elabscience Biotechnology Co., Ltd. (Wuhan, China). APC Rat Anti-Mouse CD8a, FITC Rat Anti-Mouse CD4, PerCP-Cy™5.5 Rat Anti-Mouse CD19, and PE Rat Anti-Mouse CD69 antibodies were purchased from BD Biosciences (San Jose, CA, USA). PE anti-mouse CD178 (FasL) antibody was purchased from BioLegend (San Jose, CA, USA). Reagents for detecting HBsAg-specific IgG, IgG1, and IgG2a were purchased from Jiangsu Meimian Industrial Co., Ltd. (Yancheng, China). Mouse Spleen Lymphocyte Isolation Solution Kit was supplied by Beijing Solarbio Science & Technology Co., Ltd. (Beijing, China).

### 2.2. Preparation and Characterization of GHNP

GLP was extracted and purified from *Ganoderma lucidum* according to our previously reported method [19]. In total, 34.7 mg PEG_5k_ -PCL_13k_ and 13.3 mg DOTAP were co-dissolved in 1 mL tetrahydrofuran (THF), which was rapidly injected into 2 mL deionized water under stirring at 500 rpm in a fume hood until THF evaporation yielded hybrid nanoparticle (HNP). HNP was mixed with GLP at various mass ratios of 120, 24, 12, 6, and 4 to generate GHNP120, GHNP24, GHNP12, GHNP6, and GHNP4, respectively. Particle size, zeta potential, and polydispersity index (PDI) of HNP and GHNPs were analyzed using an Optronix 919SZ analyzer (Optronix Corporation, Shanghai, China). Transmission electron microscopy (TEM) imaging of GHNP6 was conducted on a Talos F200C microscope (Thermo Fisher Scientific, Waltham, MA, USA) to observe the morphology.

### 2.3. Cell Culture

Mouse mononuclear macrophage RAW264.7 cells were procured from Wuhan Pricella Biotechnology Co., Ltd. (Wuhan, China) and cultured in DMEM complete medium containing 10% fetal bovine serum (FBS) and 1% penicillin-streptomycin. Cells were maintained at 37 °C in a humidified atmosphere with 5% CO_2_.

### 2.4. Antigen Uptake

FITC-BSA served as the model antigen. BSA@HNP, BSA@GHNP4, BSA@GHNP6, BSA@GHNP12, BSA@GHNP24, and BSA@GHNP120 formulations were prepared by mixing BSA with adjuvant nanoparticles at a fixed BSA-to-HNP mass ratio of 1:600. The mixtures were incubated at 4 °C for 30 min to ensure stable complex formation. RAW264.7 were seeded in 24-well plates (3 × 10^5^ cells/well) and cultured for 24 h. After medium removal, BSA@HNP, or BSA@GHNP formulations (0.15 μg·mL^−1^ BSA equivalent) were added to respective wells. Following 4 h incubation, cells were harvested for flow cytometry analysis of FITC-positive cell populations.

### 2.5. Cell Viability

RAW264.7 cells were seeded into 96-well plates at a density of 2 × 10^4^ cells per well and cultured for 24 h. The culture medium was then removed, and cells were treated with serum-free DMEM containing different concentrations of GHNP6. According to the experimental design, the GHNP6 concentrations were set as 0, 0.1, 1, 10, and 100 μg·mL^−1^. After 24 h of incubation, the medium was replaced with 100 μL of fresh DMEM containing 10% CCK-8 reagent. Cells were further incubated for 30 min, and the absorbance at 450 nm was measured using a microplate reader. Cell viability was calculated relative to the untreated control group.

### 2.6. Antigen Presenting Cells (APCs) Activation

To investigate the activation of APCs induced by GHNP6, RAW264.7 cells were seeded in 12-well plates at a density of 3 × 10^5^ cells per well and cultured for 24 h. Then the cells were treated with HNP or GHNP6 for 24 h, with a final equivalent HNP concentration of 8.6 μg·mL^−1^. In the Aluminum control group, the concentration of Aluminum adjuvant was set at 0.18 μg·mL^−1^. After treatment for 24 h, cells were collected and stained with fluorescence-conjugated antibodies against MHC II. The expression levels of MHC II were detected by flow cytometry. To further evaluate the role of TLR2 and TLR4 signaling in GHNP6-induced cytokine secretion, RAW264.7 cells were seeded and cultured under the same conditions. Cells were pretreated with the TLR2 antagonist CU-CPT22 (8 μM) or the TLR4 antagonist TAK-242 (0.05 μM) for 2.5 h. After pretreatment, cells were incubated with HNP or GHNP6 at an equivalent HNP concentration of 10 μg·mL^−1^ for 24 h. The cell culture medium was then centrifuged to remove cell debris, and the supernatants were collected. The level of TNF-α in the supernatant was measured using an ELISA kit.

### 2.7. Mice

Female C57BL/6 mice aged 6–8 weeks were purchased from Guangzhou Yancheng Biotechnology Co., Ltd. (Guangzhou, China). All animal experiments were conducted in full compliance with the Regulations for the Care and Use of Laboratory Animals and the Guideline for Ethical Review of Animals (China, GB/T 35892-2018) [20]. The mice were acclimated for one week before subsequent experiments. All animal experiments were approved by the Institutional Animal Care and Use Committee (IACUC) of Shenzhen University Medical School, Approval No. IACUC-202400058.

### 2.8. Animal Immunization

Hepatitis B surface antigen (HBsAg) was used for immunization. Female C57BL/6 mice were randomly divided into five experimental groups: PBS group, free HBsAg, HBsAg + Aluminum, HBsAg@HNP, and HBsAg@GHNP6. For the HBsAg@HNP and HBsAg@GHNP6 groups, the HBsAg-to-HNP mass ratio was fixed at 1:600. In the HBsAg + Aluminum group, free HBsAg was mixed with aluminum adjuvant at an HBsAg-to-Aluminum mass ratio of 1:25. Following a 7-day acclimatization period, mice were immunized intramuscularly on Days 0, 14, and 28 with different HBsAg vaccine formulations (2 μg/mouse), with 25 μL of the formulation injected into each hind leg per immunization. Body weight was monitored every 3 days throughout the experiment. Blood samples were collected from the orbital venous plexus on Days 14, 28 and 42, and serum was isolated for the detection of HBsAg-specific IgG, IgG1, and IgG2a levels by ELISA.

### 2.9. Splenocyte Activation and Cytokine Detection

On Day 42, mice were euthanized, and spleens were aseptically collected. Splenic lymphocytes were isolated using a Mouse Spleen Lymphocyte Isolation Solution Kit according to the manufacturer’s instructions. The isolated splenic lymphocytes were resuspended in complete DMEM and seeded into 12-well plates at a density of 5 × 10^6^ cells mL^−1^. Cells were then restimulated with HBsAg at a final concentration of 5 μg·mL^−1^ and incubated for 48 h at 37 °C in a humidified atmosphere containing 5% CO_2_. After restimulation, cells were collected for flow cytometry analysis. Briefly, splenic lymphocytes were washed with PBS and stained with PE anti-mouse CD178 (FasL), APC Rat Anti-Mouse CD8a, FITC Rat Anti-Mouse CD4, PerCP-Cy™5.5 Rat Anti-Mouse CD19, and PE Rat Anti-Mouse CD69 antibodies according to the manufacturers’ instructions. After staining, the cells were washed and resuspended in FACS buffer. The percentages of CD4^+^ T cells, CD8^+^ T cells, CD19^+^ B cells, CD8^+^FasL^+^ cells, CD4^+^CD69^+^ cells, CD8^+^CD69^+^ cells, and CD19^+^CD69^+^ cells were analyzed by flow cytometry. In parallel, the culture supernatants of HBsAg-restimulated splenic lymphocytes were collected after 48 h of incubation and centrifuged to remove cell debris. The levels of IFN-γ and IL-6 in the supernatants were measured using ELISA kits according to the manufacturers’ protocols. Splenocyte proliferation after HBsAg restimulation was also evaluated to assess antigen-specific lymphocyte responses.

## 3. Results and Discussion

### 3.1. Preparation and Characterization of GHNP

**Figure 2:**
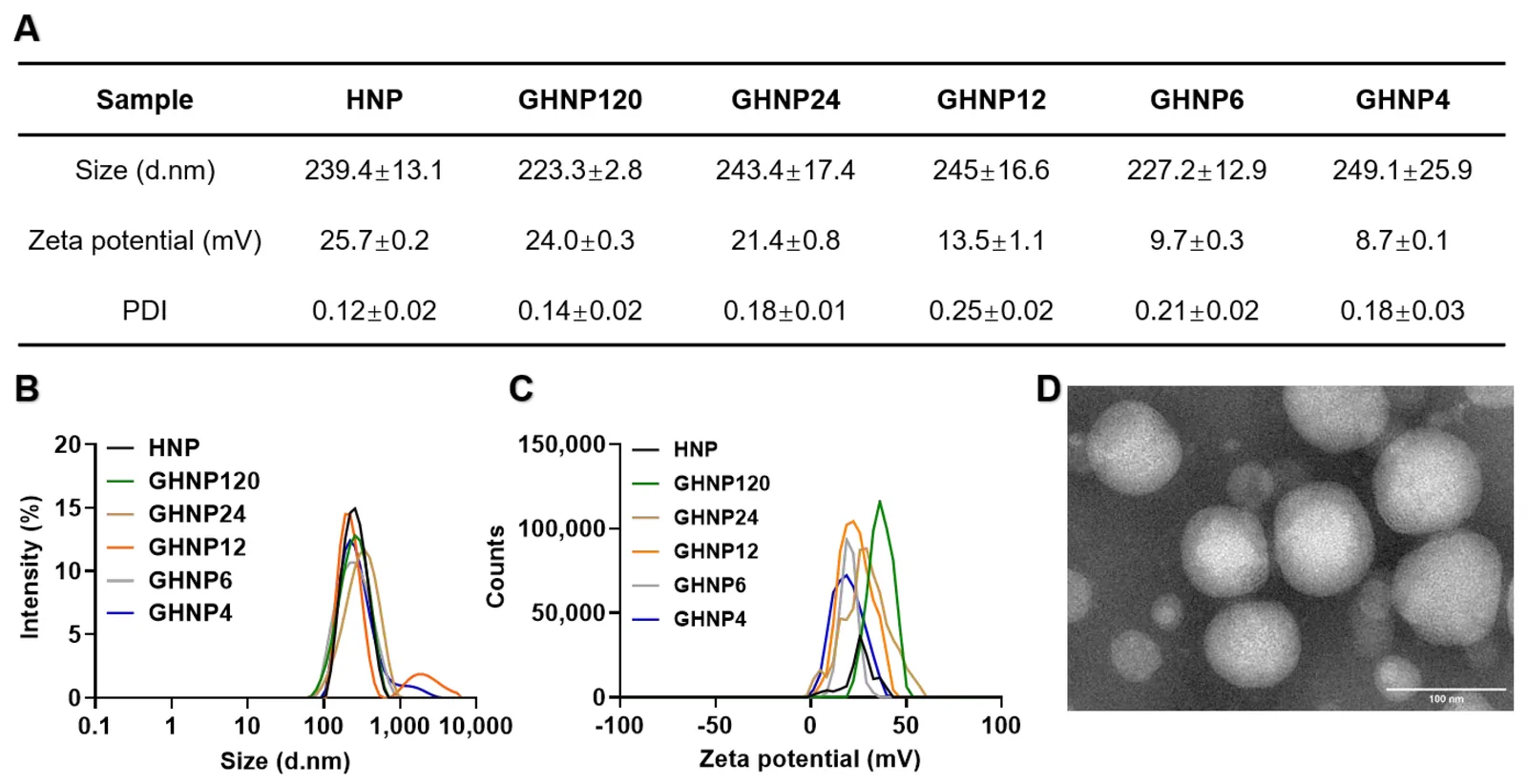
(**A**) Hydrodynamic diameter, zeta potential, and PDI of HNP, GHNP120, GHNP24, GHNP12, GHNP6, and GHNP4 measured by DLS. (**B**) Size distribution profiles of HNP and GHNP formulations. (**C**) Zeta potential distribution profiles of HNP and GHNP formulations. (**D**) Representative TEM image of GHNP6. Scale bar = 100 nm.

The purified GLP preparation had a total carbohydrate and protein content of 96.8%, consisting of 44.6% carbohydrate and 52.2% protein, and was characterized as a highly purified native glycoprotein complex [19]. As shown in Figure 2A,B, the physicochemical properties of HNP and various GHNPs were characterized to clarify the effect of GLP surface modification on nanoparticle interfacial properties and colloidal stability. DLS analysis showed that both HNP and GHNP formulations maintained average hydrodynamic diameters of approximately 200 nm, with relatively narrow size distribution profiles. This indicates that, within the tested HNP/GLP ratio range, the introduction of GLP did not disrupt the self-assembled structure of HNP or induce obvious particle aggregation. Although slight variations in particle size were observed among different GHNP formulations, the overall size changes were limited, and the PDI results further indicated acceptable dispersity for all formulations, suggesting that GLP mainly modulated the nanoparticle surface interface rather than substantially altering the colloidal stability.

Compared with particle size, zeta potential was more sensitive to GLP modification (Figure 2C). Bare HNP displayed a distinctly positive surface charge, whereas the zeta potential of GHNP formulations gradually decreased with increasing GLP content. GHNP120 and GHNP24 still retained relatively high positive charges, indicating that low-level GLP modification only partially shielded the cationic surface of HNP. When the GLP modification ratio was further increased to GHNP12, GHNP6, and GHNP4, the zeta potential decreased markedly, demonstrating that negatively charged GLP was effectively adsorbed onto the cationic HNP surface and progressively attenuated its positive charge. Notably, even at the relatively high GLP modification level of GHNP4, no charge reversal was observed, suggesting that the nanoparticles still retained a certain cationic character, which would be beneficial for subsequent antigen loading and cellular interactions.

Figure 2D confirmed that GHNP6 possessed a compact nanoparticle morphology without obvious aggregation. Taken together, these results demonstrate that GLP modification successfully regulated the interfacial properties of GHNPs, providing a suitable formulation basis for subsequent antigen delivery, cellular uptake, and immune activation studies.

### 3.2. Optimization of GLP Modification for Antigen Uptake

**Figure 3:**
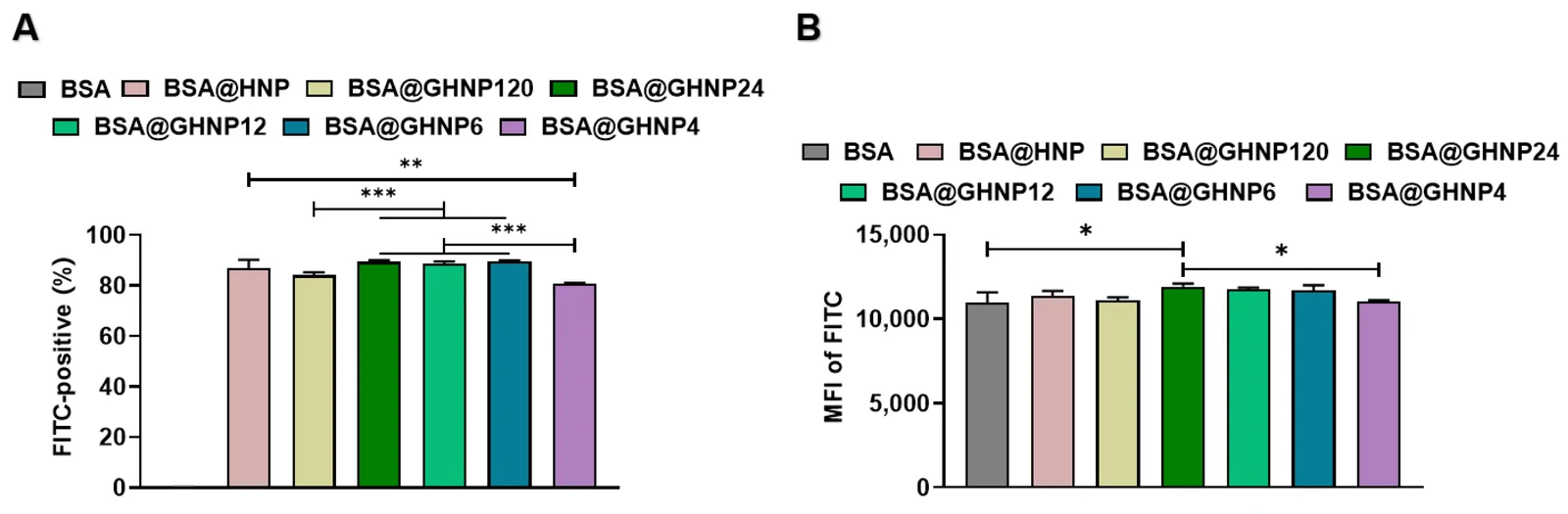
Antigen uptake efficiency of different BSA@GHNP formulations in RAW264.7 cells. (**A**) The percentage of FITC-positive cells and (**B**) the MFI of FITC-positive cells determined by flow cytometry after incubation with BSA@HNP, BSA@GHNP120, BSA@GHNP24, BSA@GHNP12, BSA@GHNP6, and BSA@GHNP4 at an equivalent BSA concentration of 0.15 μg·mL^−1^ for 4 h. (*p* < 0.05, * *p* < 0.01, ** *p* < 0.001, and *** *p* < 0.0001). Data were analyzed using one-way ANOVA.

Using FITC-BSA as a model antigen, the cellular uptake efficiency of different GHNPs was evaluated to assess antigen delivery efficiency. As shown in Figure 3, free BSA showed almost negligible cellular uptake, indicating that soluble antigen alone was poorly internalized by RAW264.7 cells. In contrast, BSA@HNP exhibited a relatively high FITC-positive cell percentage, indicating that the cationic HNP carrier could efficiently promote antigen internalization. After GLP modification, the antigen uptake efficiency showed a clear formulation-dependent pattern rather than a simple dose-dependent increase.

Among the tested formulations, BSA@GHNP24, BSA@GHNP12, and BSA@GHNP6 maintained higher FITC-positive cell percentages than the BSA@HNP and BSA@GHNP4 groups. This result suggests that appropriate GLP modification may improve the interaction between nanoparticles and cells, possibly through a combined effect of preserved cationic surface properties and GLP-mediated biological recognition. In contrast, BSA@GHNP120 did not show an obvious enhancement compared with BSA@HNP, indicating that insufficient GLP modification may be inadequate to provide additional uptake-promoting effects. Notably, when the GLP modification level was further increased to GHNP4, the FITC-positive cell percentage decreased markedly. This reduction suggests that excessive GLP adsorption may overly shield the cationic surface of HNP, thereby weakening electrostatic interactions between nanoparticles and the cell membrane and impairing antigen internalization. Therefore, GLP modification does not continuously enhance antigen uptake with increasing modification density; instead, an optimal interfacial balance is required. Therefore, GLP modification requires an appropriate interfacial balance between maintaining antigen delivery efficiency and introducing GLP-mediated immunomodulatory functionality. Considering that GLP was incorporated not only as a surface modifier but also as an immune-regulating component, GHNP6 was selected as the preferred formulation because it provided relatively high GLP incorporation while preserving efficient antigen uptake and favorable physicochemical properties. Notably, GHNP6 maintained antigen internalization efficiency despite the reduced surface charge compared with HNP, supporting its suitability for subsequent immune activation and in vivo immunization studies.

To further evaluate antigen uptake at the single-cell level, the mean fluorescence intensity (MFI) of FITC-positive cells was additionally analyzed. Unlike the percentage of FITC-positive cells, which reflects the proportion of antigen-responding cells within the population, FITC MFI provides an estimation of the relative amount of intracellular antigen associated with individual positive cells. As shown in Figure 3B, the FITC MFI values among different BSA@GHNP formulations remained comparable, indicating that GLP modification did not substantially impair the amount of antigen internalized by individual cells. These results further demonstrate that appropriate GLP modification preserves antigen delivery efficiency at both the population and single-cell levels. Therefore, GHNP6 was selected as the optimized formulation because it maintained comparable antigen uptake intensity while incorporating a higher GLP content, allowing the immunomodulatory function of GLP to be maximized without compromising antigen delivery.

### 3.3. Cell Viability

**Figure 4:**
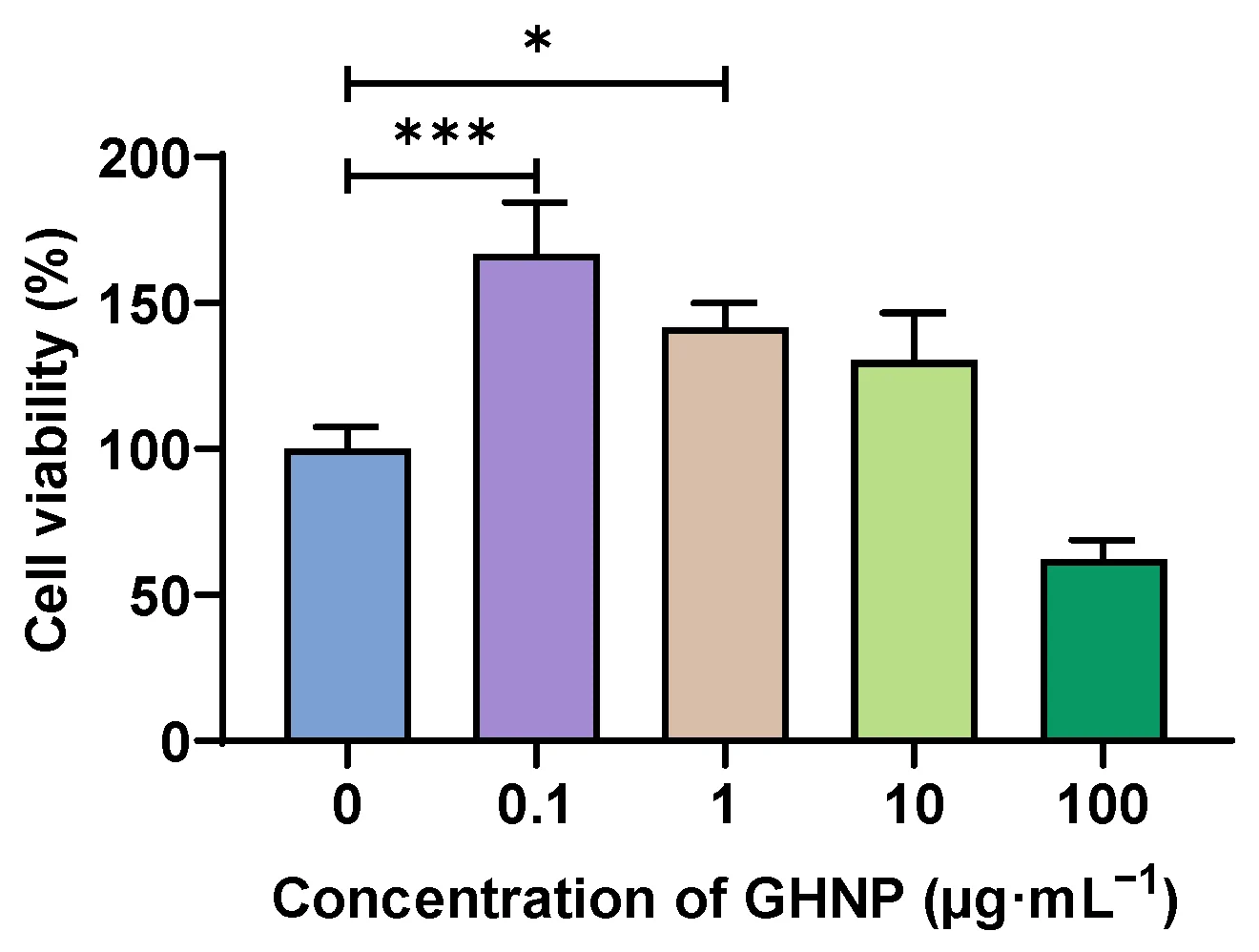
Cell viability was determined after incubation with GHNP at different concentrations for 24 h. (* *p* < 0.05 and *** *p* < 0.001). Data was analyzed using one-way ANOVA method.

The cytotoxicity of GHNP was evaluated to determine its biocompatibility and suitable concentration range for subsequent cellular experiments. As shown in Figure 4, GHNP exhibited a concentration-dependent effect on cell viability. At low and moderate concentrations, GHNP did not impair cell survival. In particular, treatment with 0.1 μg·mL^−1^ GHNP resulted in a significantly higher CCK-8 signal than the untreated control. Because the CCK-8 assay reflects overall cellular metabolic activity and is influenced by the number of viable cells, this increase was interpreted as enhanced cellular activity under the tested conditions. Cell viability remained above the control level at 1 and 10 μg·mL^−1^, indicating that GHNP maintained good biocompatibility within this concentration range. However, when the concentration was increased to 100 μg·mL^−1^, cell viability decreased markedly, indicating that excessive nanoparticle exposure may induce cytotoxic stress. This reduction may be associated with increased particle–cell membrane interactions, elevated cellular uptake burden, or concentration-dependent stimulation caused by the cationic nanoparticle interface. These results suggest that GHNP displays acceptable cytocompatibility at concentrations up to 10 μg·mL^−1^, whereas a high concentration of 100 μg·mL^−1^ may exceed the tolerable exposure range for cells. In summary, GHNP showed acceptable cytocompatibility at concentrations up to 10 μg·mL^−1^ under the tested conditions, supporting the selection of this concentration range for subsequent immune activation assays.

### 3.4. APC Activation and TLR2/4 Receptor Validation

**Figure 5:**
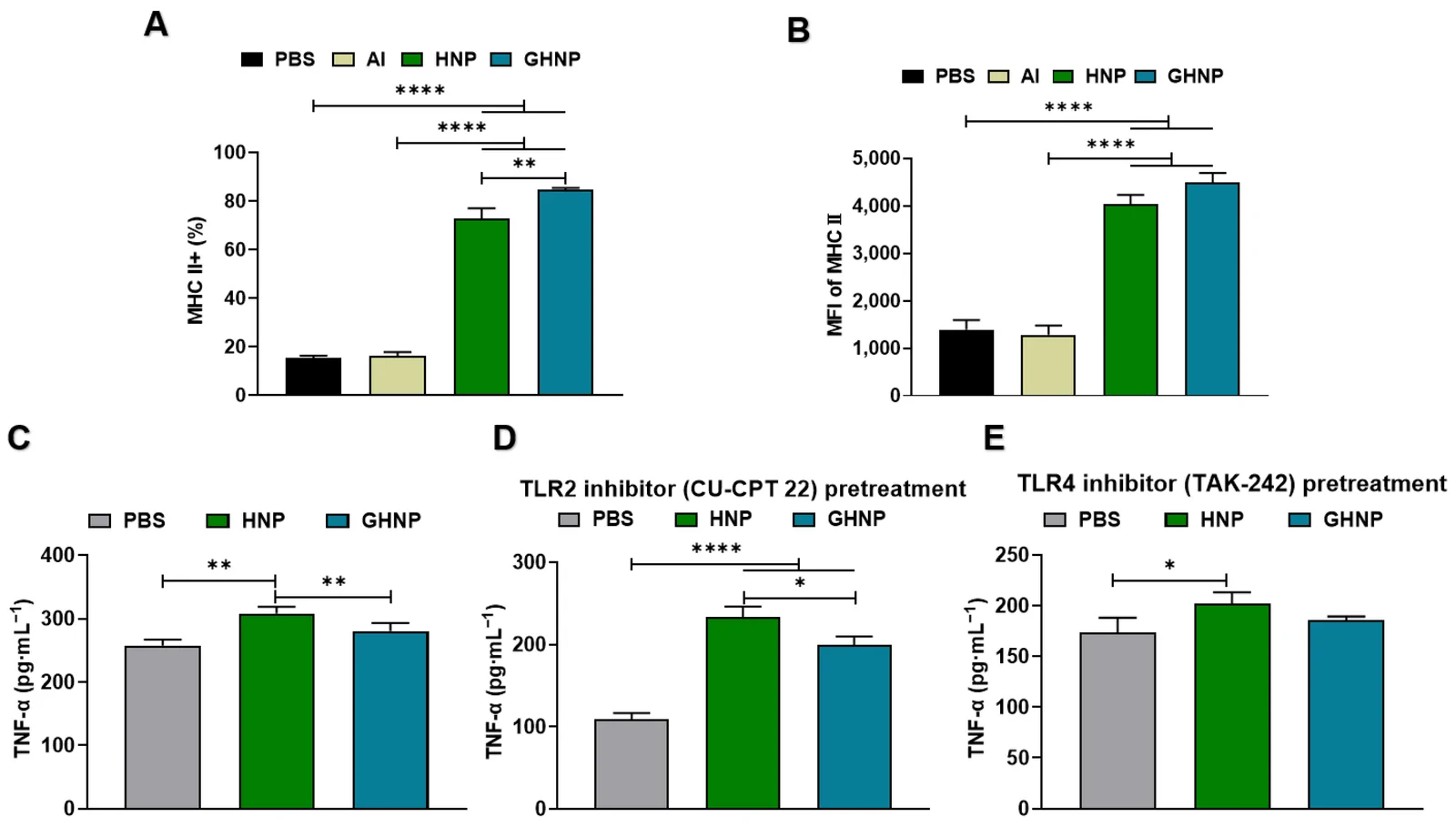
(**A**) The percentage of MHC II positive cells and (**B**) the MFI determined by flow cytometry after incubation with PBS, Aluminum, HNP, and GHNP for 24 h. The equivalent HNP concentration was 8.6 μg·mL^−1^ and the Aluminum concentration was fixed at 0.18 μg·mL^−1^. (**C**) TNF-α secretion in RAW264.7 cells after incubation with PBS, HNP, or GHNP6 for 24 h. (**D**) TNF-α secretion in RAW264.7 cells pretreated with the TLR2 antagonist CU-CPT22 (8 μM) or (**E**) the TLR4 antagonist TAK-242 (0.05 μM) for 2.5 h, followed by incubation with HNP or GHNP for 24 h. The final HNP concentration was 10 μg·mL^−1^. (* *p* < 0.05, ** *p* < 0.01 and **** *p* < 0.0001). Data was analyzed using one-way ANOVA method.

To evaluate whether GHNP could enhance antigen-presenting cell activation, MHC II expression was analyzed by flow cytometry after incubation with different formulations. As shown in Figure 5A, the PBS and Aluminum groups induced low levels of MHC II-positive cells. In contrast, HNP treatment markedly increased the percentage of MHC II-positive cells, indicating that nanoparticle-mediated delivery could promote APC activation and antigen-presenting capacity. Notably, GHNP further enhanced the proportion of MHC II-positive cells compared with HNP. This result suggests that GLP modification did not simply preserve the immune-activating effect of the HNP carrier, but provided an additional stimulatory contribution. The enhanced MHC II expression may be attributed to the combined effect of nanoparticle-facilitated antigen delivery and GLP-mediated immunomodulation, thereby promoting APC maturation and antigen presentation. Consistent with the change in MHC II-positive cell frequency, the MFI of MHC II was also markedly increased in the HNP and GHNP groups compared with PBS and Aluminum groups (Figure 5B). This indicates that nanoparticle formulations not only increased the number of MHC II-expressing cells but also enhanced the expression level of MHC II on individual cells. Compared with the Aluminum group, the HNP formulation substantially improved APC activation, while GLP modification further strengthened the antigen-presenting phenotype. This provides an important cellular basis for the enhanced immune responses induced by GHNP in subsequent immunization studies.

After confirming that GHNP enhanced MHC II expression, we further investigated whether TLR2/4 participated in nanoparticle-induced APC activation. TNF-α is a representative pro-inflammatory cytokine mainly produced by activated macrophages and other innate immune cells. As a downstream effector of TLR-mediated NF-κB signaling, TNF-α reflects early macrophage activation and inflammatory cytokine production [21,22]. Functionally, TNF-α contributes to the amplification of innate immune responses by promoting inflammatory mediator release, immune cell recruitment, and communication between innate and adaptive immunity [23,24]. Therefore, TNF-α secretion was selected as a functional readout to evaluate whether HNP- or GHNP-induced APC activation involved TLR2/4-associated inflammatory signaling. TNF-α secretion was first measured in RAW264.7 cells after incubation with PBS, HNP, or GHNP. TNF-α secretion was first measured in RAW264.7 cells after incubation with PBS, HNP, or GHNP. As shown in Figure 5C, HNP treatment significantly increased TNF-α production compared with the PBS control, indicating that the nanoparticle carrier itself could stimulate macrophage activation through direct membrane interactions. GHNP also maintained an elevated TNF-α level, although the response was slightly lower than that induced by HNP. This reduction may be associated with partial shielding of the cationic HNP surface by GLP, which attenuates non-specific electrostatic interactions with the cell membrane. To clarify the involvement of specific pattern recognition receptors, RAW264.7 cells were pretreated with the CU-CPT22 or the TAK-242 before nanoparticle stimulation. After TLR2 inhibition, TNF-α secretion was markedly reduced in both HNP and GHNP groups compared to their respective non-inhibited conditions (Figure 5D). Notably, upon TLR2 blockade, TNF-α levels in the HNP and GHNP groups decreased by 24.2% and 28.8%, respectively, compared with their corresponding non-inhibited groups. The more pronounced reduction observed in the GHNP group suggests that GLP modification may enhance the involvement of TLR2-associated signaling in nanoparticle-induced macrophage activation, shifting the immune activation mechanism from non-specific membrane perturbation toward a more receptor-associated pathway. After TAK-242 pretreatment, TNF-α levels in the HNP and GHNP groups were reduced by 34.6% and 33.8%, respectively, compared with their corresponding non-inhibited groups (Figure 5E). However, the difference between the HNP and GHNP groups became less pronounced under TLR4-blocked conditions. Together with the MHC II expression results, these findings demonstrate that GHNP can promote APC activation and antigen-presenting phenotype formation. The TLR2/4 inhibition experiments further indicate that TLR2/4 signaling are involved in GHNP-mediated immune activation to some extent. Therefore, GHNP does not function solely as a delivery carrier; rather, GLP modification provides receptor-associated immunomodulatory activity, supporting its potential role as a nanoparticle-based vaccine adjuvant.

### 3.5. In Vivo Immunization

**Figure 6:**
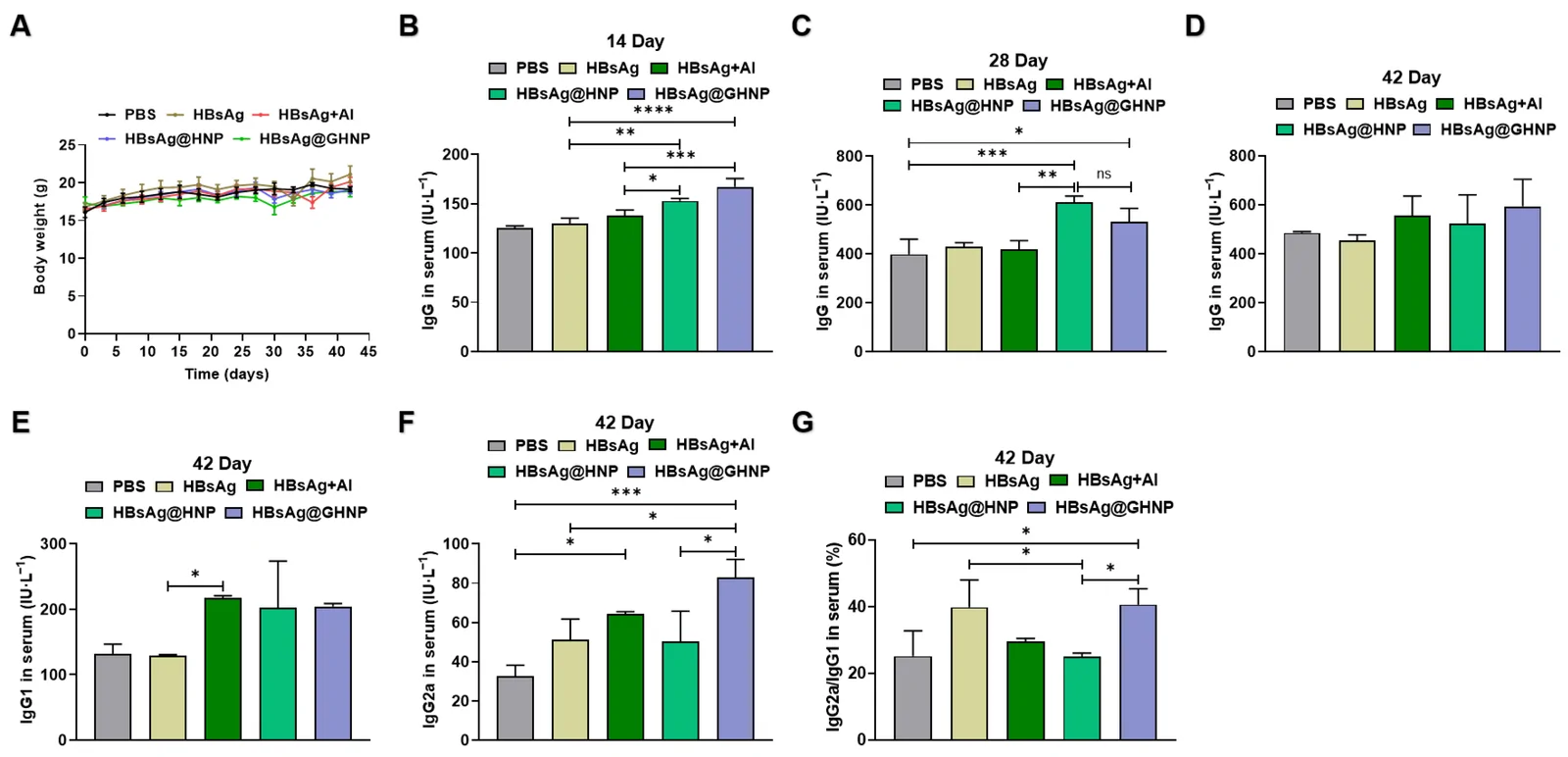
(**A**) Body weight of immunized mice every 3 days throughout immunization. (**B**–**D**) Serum levels of HBsAg-specific IgG on Days 14, 28, and 42. (**E**) Serum levels of HBsAg-specific IgG1 on Day 42. (**F**) Serum levels of HBsAg-specific IgG2a on Day 42. (**G**) The HBsAg-specific IgG2a/IgG1 ratio in serum on Day 42. (* *p* < 0.05, ** *p* < 0.01, *** *p* < 0.001, and **** *p* < 0.0001). Data was analyzed using one-way ANOVA method.

Body weight was monitored throughout the immunization period to evaluate the safety of different formulations. As shown in Figure 6A, mice in all groups gradually gained weight during the 42-day experiment, and no obvious weight loss was observed after repeated intramuscular immunization. The body weight curves of the HBsAg@HNP and HBsAg@GHNP groups were comparable to those of the PBS, free HBsAg, and HBsAg + Al groups. These results suggest that HBsAg@GHNP did not induce apparent systemic toxicity or adverse effects under the current immunization regimen, supporting its acceptable in vivo tolerability.

Serum HBsAg-specific antibody responses were then evaluated to determine the humoral immune efficacy of different vaccine formulations. As shown in Figure 6B, HBsAg@GHNP showed a relatively higher mean IgG level on Day 14 compared with the other groups, although the difference was modest. This result suggested a tendency toward enhanced early HBsAg-specific antibody responses. On Day 28, both HBsAg@HNP and HBsAg@GHNP maintained elevated IgG levels compared with PBS and free HBsAg groups, demonstrating that nanoparticle-based formulations enhanced antibody production during the booster phase (Figure 6C). At Day 42, the GHNP group showed the highest mean total IgG level among all groups, although this difference did not reach statistical significance. To further characterize the quality of the HBsAg-specific humoral response, antigen-specific IgG1 and IgG2a subclass levels were analyzed, and the IgG2a/IgG1 ratio was calculated. As shown in Figure 6E–G, HBsAg@GHNP exhibited a higher HBsAg-specific IgG2a/IgG1 ratio than HBsAg@HNP and HBsAg + Al, suggesting a shift toward a more Th1-associated antigen-specific humoral response. Notably, the total HBsAg-specific IgG levels in the free HBsAg and HBsAg + Al groups were not significantly different from those in the PBS group at the examined time points. This relatively weak antibody response may be associated with the antigen dose and immunization regimen used in the present study. Therefore, the total IgG results should be interpreted together with the IgG subclass profile and antigen-specific cellular immune responses rather than as a standalone indicator of immunization efficacy. The minor variation observed in the PBS group among different time points may be attributed to assay-related variability at low background antibody levels. Therefore, these fluctuations were considered technical variation rather than biologically meaningful changes in HBsAg-specific antibody production.

### 3.6. Splenocyte Activation and Cytokine Detection

**Figure 7:**
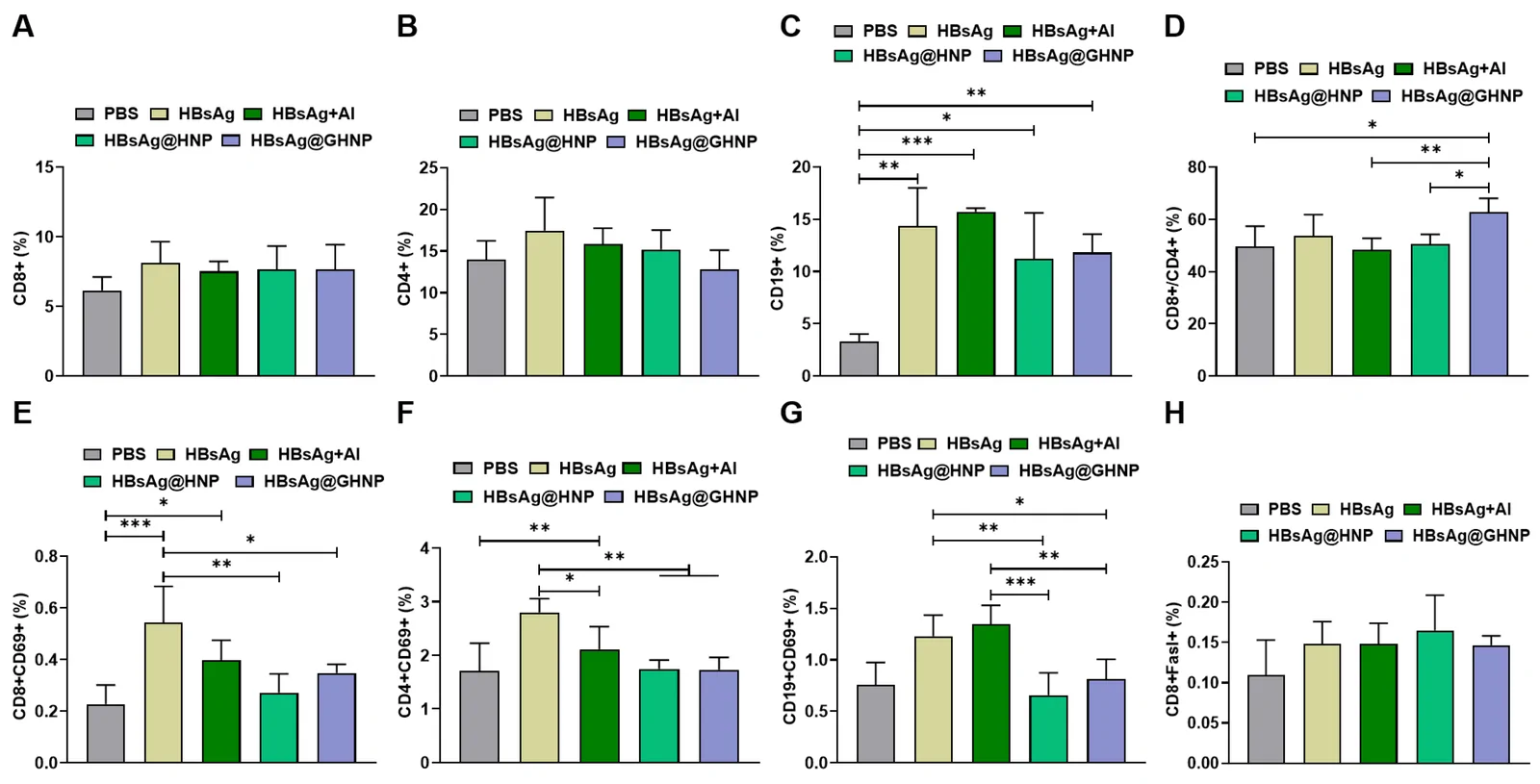
(**A**) Percentage of CD8+ T cells. (**B**) Percentage of CD4+ T cells. (**C**) Percentage of CD19+ B cells. (**D**) Ratio of CD8+/CD4+ T cells. (**E**) Percentage of CD8+CD69+ T cells. (**F**) Percentage of CD4+CD69+ T cells. (**G**) Percentage of CD19+CD69+ B cells. (**H**) Percentage of CD8+FasL+ T cells. (* *p* < 0.05, ** *p* < 0.01, and *** *p* < 0.001). Data was analyzed using one-way ANOVA method.

Splenic lymphocyte populations were further analyzed by flow cytometry to assess cellular immune responses after immunization. As shown in Figure 7A,B, the percentages of CD8+ and CD4+ T cells showed only moderate changes among the different groups, indicating that the vaccine formulations did not markedly disturb the overall distribution of major T-cell subsets. However, the CD8+/CD4+ ratio was increased in the HBsAg@GHNP group, especially after HBsAg restimulation (Figure 7C), suggesting that the GHNP formulation may favor a relatively stronger CD8+ T-cell-associated response. The proportion of CD8+FASL+ cells showed no obvious difference among the groups (Figure 7D), indicating that the current immunization regimen did not strongly induce FASL-associated cytotoxic activation at the tested time point. In contrast, CD19+ B-cell percentages were increased in the antigen-immunized groups, particularly in the HBsAg + Al and nanoparticle-formulated groups (Figure 7E), suggesting that HBsAg immunization promoted B-cell expansion or enrichment in the spleen. In addition, CD69 expression analysis showed that antigen-containing formulations increased the proportions of activated CD8+CD69+, CD4+CD69+, and CD19+CD69+ lymphocyte subsets to varying degrees (Figure 7F–H), indicating that different adjuvant systems induced distinct lymphocyte activation patterns rather than uniformly enhancing all immune cell populations.

**Figure 8:**
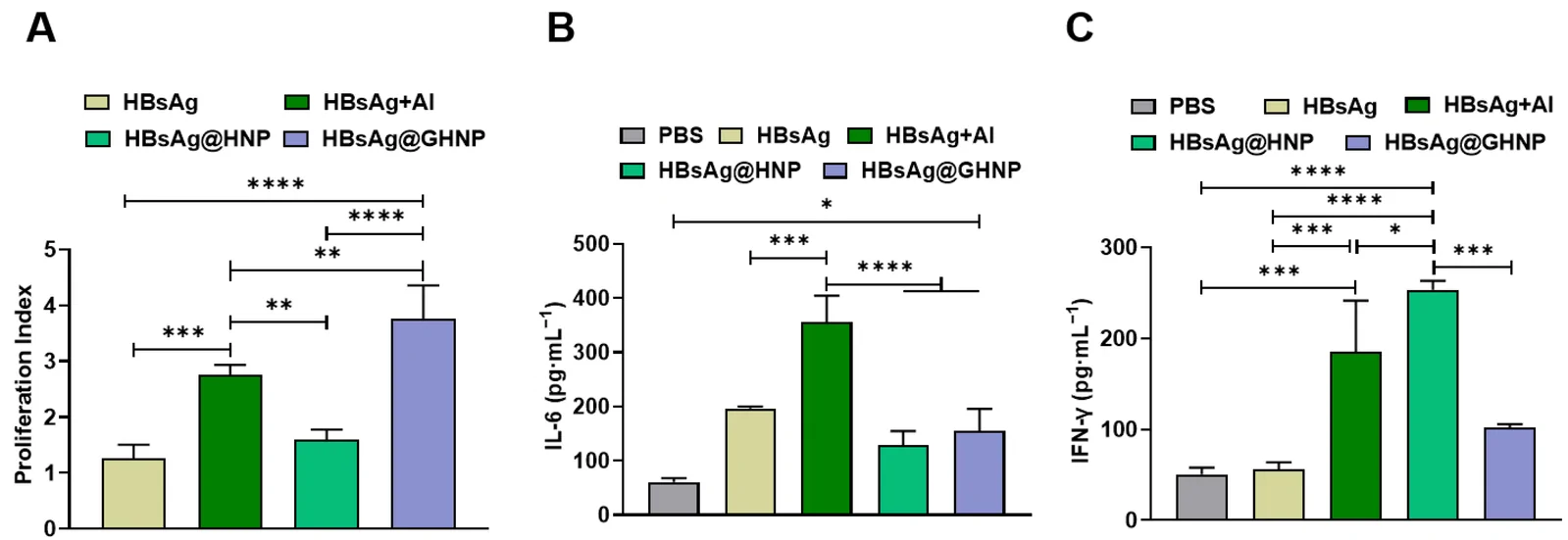
(**A**) Splenocyte proliferation index after HBsAg restimulation. (**B**,**C**) Levels of IL-6 and IFN-γ in the culture supernatants of HBsAg-restimulated splenic lymphocytes, as determined by ELISA. (* *p* < 0.05, ** *p* < 0.01, *** *p* < 0.001, and **** *p* < 0.0001.) Data was analyzed using one-way ANOVA method.

To further assess antigen-specific cellular immune function, splenic lymphocytes were isolated on Day 42 and restimulated with HBsAg in vitro. As shown in Figure 8A, HBsAg@GHNP induced the highest splenocyte proliferation among the tested groups, suggesting that GHNP effectively promoted antigen-specific lymphocyte expansion after recall stimulation. Cytokine secretion in the culture supernatants was also measured. HBsAg + Al induced the highest IL-6 production, indicating a strong IL-6-associated inflammatory or humoral immune response (Figure 8B). In contrast, HBsAg@HNP and HBsAg@GHNP induced relatively lower IL-6 levels than HBsAg + Al, suggesting that nanoparticle-based formulations generated a different cytokine profile rather than simply amplifying IL-6 secretion. IFN-γ secretion showed a distinct pattern, with HBsAg@HNP inducing the highest IFN-γ level, whereas HBsAg@GHNP produced a moderate but still elevated IFN-γ response compared with PBS and free HBsAg groups (Figure 8C). Although the IFN-γ for HBsAg@GHNP decreased compared to HBsAg@HNP, but moderately increased than the HBsAg group. The enhanced response of HBsAg-restimulated splenocytes (Figure 8A) indicated that GHNP promoted stronger antigen-responsive cellular activation under the tested conditions. However, the CCK-8-based assay reflects overall cellular metabolic activity and viable cell number, and cannot directly identify the specific immune cell populations contributing to this response. Therefore, these results suggest enhanced activation of antigen-responsive splenocytes. Furthermore, the combination of enhanced splenocyte activation with regulated cytokine production suggests that GHNP may promote a balanced immune response with Th1-associated characteristics. Compared with aluminum adjuvant, GHNP represents an alternative immunomodulatory strategy by integrating nanoparticle-mediated antigen delivery with GLP-mediated immune regulation.

These results indicated that HBsAg@GHNP enhanced antigen-specific splenocyte proliferation and supported both humoral and cellular immune responses, while maintaining an acceptable in vivo safety profile.

## 4. Conclusions

We developed a *Ganoderma lucidum* polysaccharide-modified hybrid nanoparticle adjuvant, GHNP, to improve the immune quality of HBsAg-based hepatitis B vaccination. Through facile surface adsorption of GLP onto the cationic PEG-PCL/DOTAP nanoparticle, GHNP achieved balanced interfacial properties, maintaining efficient antigen uptake while introducing GLP-mediated immunomodulatory activity. In vitro, GHNP showed favorable cytocompatibility, enhanced antigen internalization, promoted MHC II upregulation in APCs, and activated TLR2/4-associated inflammatory signaling, indicating that it functions not only as an antigen delivery carrier but also as an immune-modulating adjuvant. However, the relatively modest total IgG responses observed under the current immunization conditions highlight the need for further optimization of the antigen dose and immunization regimen through systematic dose-response studies. In vivo, HBsAg@GHNP induced robust HBsAg-specific antibody responses, increased the IgG2a/IgG1 ratio compared to HBsAg@HNP and HBsAg + Al, and promoted antigen-specific splenocyte proliferation with moderately enhanced IFN-γ and IL-6 secretion. Collectively, GHNP establishes a natural polysaccharide-functionalized nanoadjuvant platform that integrates nanoparticle-assisted antigen delivery with GLP-driven immune regulation, providing a promising strategy to overcome the Th2-biased limitation of conventional aluminum-adjuvanted hepatitis B vaccines and to enhance cellular immune potency.

## Data Availability

The data presented in this study are available on request from the corresponding author due to reasonable request.
